# Molecular and Phytochemical Variability of Common Juniper (*Juniperus communis* L.) in the Central Balkans Reveals Differentiation of Populations

**DOI:** 10.3390/plants15081266

**Published:** 2026-04-20

**Authors:** Nemanja Rajčević, Tanja Dodoš, Peđa Janaćković, Ljubodrag Vujisić, Petar D. Marin

**Affiliations:** 1 Faculty of Biology, University of Belgrade, Studentski trg 16, 11000 Belgrade, Serbia; tanjadodos@bio.bg.ac.rs (T.D.); pjanackovic@bio.bg.ac.rs (P.J.); pdmarin@bio.bg.ac.rs (P.D.M.); 2 Faculty of Chemistry, University of Belgrade, Studentski trg 16, 11000 Belgrade, Serbia; ljubaw@chem.bg.ac.rs

**Keywords:** *Juniperus communis* var. *communis*, Cupressaceae, essential oil, inter-simple sequence repeats, multivariate statistical analysis, chemophenetics

## Abstract

*Juniperus communis* is the juniper with the widest geographical distribution, owing to its high ecological valence. Nevertheless, there is only a limited number of studies of its phenotypic and molecular variability. In this study, we coupled leaf essential oil (EO) composition with molecular and environmental data to better understand this species’ distribution and variability in the central Balkans. EOs were obtained by simultaneous hydrodistillation and extraction, and analysed using GC coupled with MS and FID detectors. For molecular analysis, inter-simple sequence repeats (ISSR) using five primers were analysed. Three chemotypes were most abundant in the study area: sabinene, an intermediate chemotype, and α-pinene. Several additional chemotypes were also identified. In total, 118 compounds present above 0.05% were detected and identified. Monoterpene hydrocarbons dominated the EO composition (43.8–79.1%). Multivariate analyses showed separation of populations from north to south. ISSRs yielded 78 polymorphic bands. Three genetic pools could also be identified that roughly correspond to this distribution, though data is not completely congruent with chemophenetic. Results indicate high genetic diversity, with high gene flow between populations, but also certain differentiation of populations.

## 1. Introduction

*Juniperus communis* L. (Cupressaceae) is one of more than sixty taxa belonging to the genus *Juniperus*, but it is the only one with an almost holarctic distribution. It grows on dry, rocky soils from sea level in northern Eurasia and North America to well above the upper forest limit in mountain ranges at lower latitudes [1,2]. Additionally, it can be found across different soils and plant communities. This high ecological valence is based on high genetic diversity within this taxon and may also be attributed to high phenotypic plasticity. Seven varieties have been described so far: the typical *J. c.* var. *communis* with upright habitus, prostrate *J. c.* var. *saxatilis*, *J. c.* var. *depressa*, and *J. c.* var. *megistocarpa*, and *J. c.* var. *procumbent*, *J. c.* var. *nipponica*, *J. c.* var. *jackii*, and *J. c.* var. *charlottensis* [1].

Essential oils (EOs) from leaves have long been studied as useful chemophenetic markers, and numerous studies have shown that they do not vary significantly with environmental parameters [1,3,4,5,6]. Juniper species store essential oils in resin ducts in leaves and branches, so their chemical composition is quite stable and does not change significantly during storage of the plant material [7]. Common juniper leaf EOs show clear domination of monoterpenic compounds, and several chemotypes have been identified so far [3,4,5,7]. Aside from α-pinene, commonly found in conifers, sabinene and δ-3-carene have been detected at high percentages to date [4]. A previous study of leaf EO in the western Balkans [4] showed a clear separation between populations under Mediterranean and more continental climates.

While previous studies using different chloroplast and nuclear sequences did not show regional separation in common juniper, the significant ecological valence should not be attributed solely to phenotypic plasticity. Several studies using multilocus molecular markers (RFLP, RAPD, and AFLP) and microsatellites (SSR) have shown that genetic variability in this taxon is high, but provide insufficient evidence for the geographical separation of populations [8,9,10,11,12]. Inter-Simple Sequence Repeats (ISSRs) have been used to study genetic diversity in crops and have been shown to be useful, as cheap and reliable markers, for the study of genetic structure. Unlike SSRs, which focus on specific loci and the variability in the number of repeats, ISSRs target multiple loci across microsatellite regions, thereby sampling a wider range of genetic variation [13].

The aim of this study is to investigate the chemical and genetic variability of *Juniperus communis* var. *communis* from the central Balkans that would serve as bases for a deeper insight into the genetic and phenetic potential of this ecologically and economically important species. Different environmental parameters will be considered to help explain the observed phenotypic variability (Table 1).

## 2. Results

### 2.1. Essential Oil Composition

Chemical analysis of 134 obtained leaf essential oils yielded over 150 different compounds. Table 1 shows 118 compounds present at trace levels (0.05 < x < 0.1%) in more than one population; the remaining compounds were excluded from the analysis due to their low abundance. The identified compounds account for 97.0–98.9% of the total oil. While a high number of compounds were detected overall, individual samples contained 53–91 compounds, indicating substantial variability in EO composition. Additionally, many compounds show significant variability within and between populations, suggesting the existence of chemotypes in the sample.

Most of the oils were dominated by monoterpenes (69.8–79.1%). An exception was the population of Mt. Kozjak, where the ratio of monoterpenes to sesquiterpenes was approximately 43.8%:45.0%. Additionally, hydrocarbons dominated over oxygenated compounds across all samples. Diterpene concentrations were usually around 1–2% of the total oil, except for Mt. Kozjak, which was 8.8%.

Two compounds were the most abundant in the EO composition: α-pinene and sabinene. These two compounds accounted for about half of the essential oil composition, though they were not always present at the same percentage across individuals. Aside from these compounds, limonene, terpinen-4-ol, germacrene D, and germacrene B were also present in higher concentrations. Eighteen compounds, which were present at 0.5% or higher on average, were not correlated, and showed a normal distribution, were used in the statistical analyses.

Principal component analysis (Supplemental Figure S1) shows separation of all samples based on only three components: α-pinene and sabinene on the first axis (76.4%) and limonene on the second (14.1%). Based on the abundance ratio of α-pinene and sabinene, mixture analysis suggests the existence of three distinct groups (the lowest Akaike IC value). Most individuals could be grouped into three major groups: high α-pinene (first group), high sabinene (second group), and intermediate α-pinene and sabinene (third group). However, several individuals could not be assigned to any of the above groups. These results suggested the existence of at least three putative chemotypes, though several more chemotypes could be present, albeit in low numbers (Supplemental Figures S2–S4).

**Figure 1:**
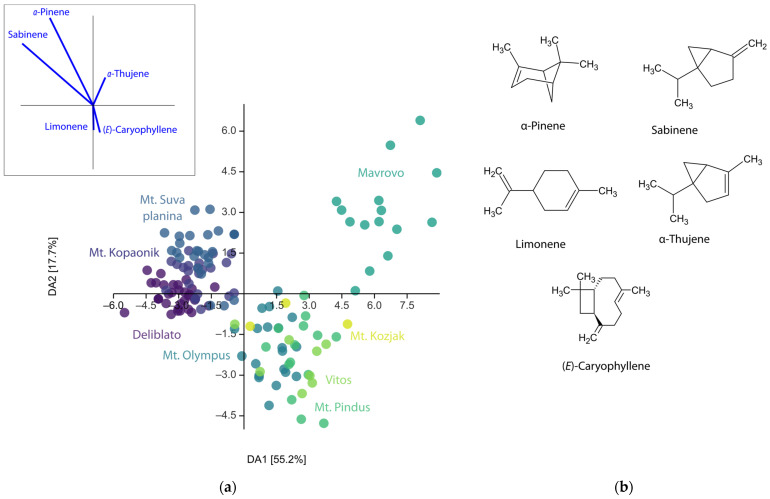
(**a**) Discriminant analysis scatter plot of eighteen components of *Juniperus communis* L. var. *communis* leaf essential oil. Populations were used as a priori groups; projection of the original axes (variables) onto the scattergram showing five components that contribute the most on the separation are given in the upper left-hand side corner; (**b**) Structural formulae of five most important terpenes according to DA.

While principal component analysis failed to separate populations based on EO composition, discriminant analysis (DA) with predefined groups as populations showed clear separation by geographic location (Figure 1). The first two eigenvectors of DA account for 72.9% of the total variability and show clustering of populations into three distinct groups. Cluster A consists of three populations from Serbia (Deliblato, Mt. Kopaonik, and Mt. Suva planina); individuals from the Mavrovo population formed cluster B, while all Greek populations with Mt. Kozjak clustered together. The confusion matrix shows that 81.3% of the individuals were correctly assigned to their groups, suggesting strong support for the population separation (group assignment was cross-validated by a leave-one-out cross-validation (jackknifing) procedure). Since Mt. Kozjak population was really small, individuals were not assigned a priori to any group, but were marked for the discriminant linear classifier and placed on a scatter plot after the discriminant analysis. Results of the DA were checked using MANOVA, which confirmed statistical significance in differentiation of populations based on EO composition (F = 13.66, *p* = 2.3 × 10^−132^). Post hoc Hotelling’s test with Bonferroni-corrected *p*-values confirmed the separation of most populations. In only seven population pairs, Hotelling’s test failed to detect statistically significant differences. Namely, Mt. Kozjak, Vitos, and Mt. Pindus had similar EO profiles, as did Mt. Olympus, Vitos, and Mt. Pindus. The population of Mavrovo was also not statistically different from that of Mt. Pindus.

**Figure 2:**
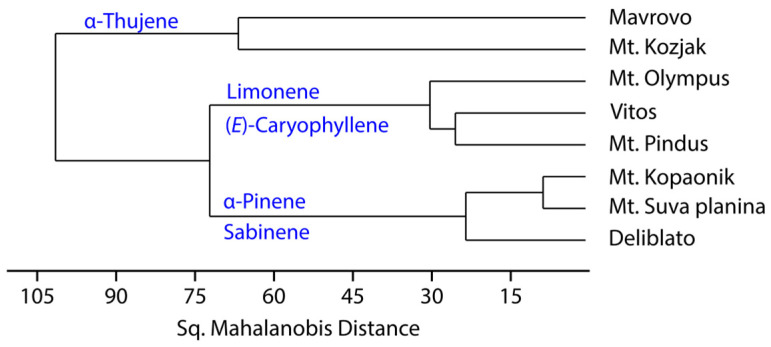
Hierarchical cluster analysis dendrogram based on eighteen components of *Juniperus communis* L. var. *communis* leaf essential oil.

Hierarchical cluster analysis (HCA) using sq. Mahalanobis distances produce somewhat different results (Figure 2). Populations were grouped by region, with those from Serbia and Greece showing a more similar composition than those from Northern Macedonia. However, given that the population Mt. Kozjak consisted of only three individuals, its positioning may be a sampling artifact resulting from the limited sample size and should be interpreted with caution.

### 2.2. Molecular Analysis

Seventeen different ISSR markers were tested. Out of these, five with the most variable bands were selected. Five selected ISSR markers yielded 78 bands across 74 individuals from eight localities. All ISSRs produced multiple polymorphic bands. The PIC values for this marker, which can reach 0.5, ranged from 0.249 to 0.334. All of the primers had low-to-moderate PIC values (0.3–0.4). (Table 2).

**Figure 3:**
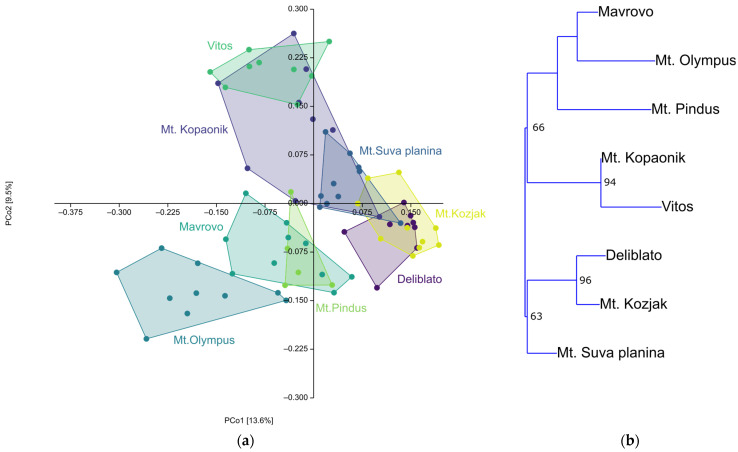
Statistical analysis of 78 polymorphic bands obtained by five ISSR markers for eight populations of *Juniperus communis* var. *communis* from the Balkans. (**a**) Principal Coordinate Analysis (Jaccard’s distances); (**b**) Neighbor-joining (Nei’s genetic distances) based on ISSR markers. Bootstrap values above 60% are written in the nodes.

Analysis of molecular variance (AMOVA) showed that most of the genetic variability (73.0%) lies within populations, while 27.0% lies between populations, suggesting moderate genetic differentiation between populations. Principal coordinate analysis using Jaccard distances (Figure 3a) accounts for 22.9% of the variability on the first two eigenvectors, separating all individuals into two major groups, with some separation of populations within those groups. These results are also confirmed by the neighbour-joining tree (NJ), which formed two major clades (Figure 3b). While most individuals from each population clustered closely together, not all did, so some populations showed higher genetic diversity than others. However, some of the NJ clades lacked statistical support.

According to these analyses, two distinct clusters were formed. Populations from eastern Serbia (Deliblato, Mt. Suva planina) and Mt. Kozjak from North Macedonia were separated from the rest. Within the second cluster, two groups could be separated: one consisting of Mt. Kopaonik (western Serbia) and Vitos (northern Greece), and the other encompassing the remaining Greek population and one population from North Macedonia.

**Figure 4:**
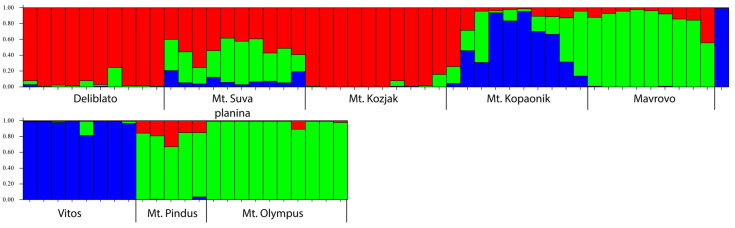
Structure analysis of the 78 polymorphic ISSR bands obtained by five markers for eight populations of *Juniperus communis* var. from the Balkans. K = 3. Individuals grouped by populations.

The structure analysis identified geographic structuring of populations. Based on the Bayesian Information Criterion (BIC), three genetic pools could be identified. Upon closer inspection, there is no strict separation of populations, and the results suggest previous exchange of genetic material, since all individuals contained to a certain degree genetic material from all three pools (Figure 4). Mantel test did not confirm any correlation between geographic proximity and genetic structure. This could be due to the relatively recent nature of these populations, which may have been influenced by human activities or a consequence of recent geographic expansion after the last glaciation maximum.

### 2.3. Correlation with the Environmental Parameters

A linear correlation test between bioclimatic data and elevation showed no significant correlation with most individual components of the essential oil. There were, however, three exceptions: *p*-cymene with the mean temperatures of the wettest and driest quarters (R = −0.6 and 0.7, respectively), premnaspirodiene with temperature annual range (R = 0.6), and *δ*-cadinene with the precipitation of coldest quarter (R = 0.6). However, the Mantel and partial Mantel tests did not detect any correlation between EO composition and bioclimatic parameters (R= −0.33, *p* = 0.9), even after controlling for geographical region (R= −0.32, *p* = 0.88). Additionally, there was no significant correlation between EO composition and the geographic proximity of populations (R = 0.21, *p* = 0.18).

## 3. Discussion

Both phytochemical and molecular analyses confirm the presence of high genetic and phenetic diversity in *Juniperus communis* var. *communis*, which is consistent with its wide geographical distribution. Essential oil composition identified at least three chemotypes, though additional chemotypes may be present at low frequencies. Furthermore, all these chemotypes were universally present across the analysed populations, though some population differentiation based on the predominance of certain chemotypes could be discerned. For example, populations from Serbia are somewhat separated from other populations by a high prevalence of the sabinene chemotype, while the Greek populations are the opposite, with lower *α*-pinene and sabinene in the EO composition. The Mavrovo population differed from the rest in having higher *α*-thujene concentrations. When compared with previous results, the sabinen chemotype was the most prevalent in the northwestern Balkans, followed by the intermediate and *α*-pinene chemotypes [4]. The literature reports higher *α*-pinene levels in populations from Mt. Olympus [15], which was also observed in the present results, but only at the population level. At the individual level, most individuals show an intermediate chemotype, with sabinene present at higher concentrations. High levels of *α*-pinen (above 50%), previously found in populations from Corsica, Sardinia, and Hungary [15,16,17], were present in only 12 individuals in the present study, making the true α-pinen chemotype rare in the analysed sample. Since sabinene is a compound used in the flavouring and perfume industries, as well as a part of complex biofuels, these results are significant because they identify central Balkan populations rich in this compound, which is, so far, available only from plant material as its biosynthesis is too costly [15]. While there are no studies on the variability of sabinene in junipers under the influence of environmental parameters, a study on the yield of sabinene in *Piper nigrum* L. found that sabinene is influenced by precipitation and light intensity [16]. However, correlation tests did not show correlations between essential oil components or profiles with bioclimatic parameters and altitude, suggesting that if there is an environmental influence on the EO profile, it is either not simple, or it is not between climate and EO profile, but could be something different, i.e., predation by certain herbivores [17,18,19,20] or phytopathogens [21]. While this study did not take into account the substratum, it is apparent that it did not influence the composition of leaf EO, since populations that grow on different substrata and at different altitudes (e.g., Deliblato and Mt. Suva planina) share a similar EO profile.

While essential oil profiles yielded separation of populations based on individual samples’ composition, molecular markers indicated three potential genetic pools that could indicate several refugia during the last glacial maximum. The Mantel test did not show any correlation between genetic composition and geographic distances, indicating a complex natural history of the studied area. However, these results need to be verified using additional molecular markers that could capture the species’ genetic variability better. Previous investigations of the genetic diversity of *Juniperus sabina* used ISSR markers [22]. This investigation has shown that ISSR markers are useful in capturing genetic diversity in this species. At the same time, a previous investigation of common juniper using SSR markers identified three genetic pools in the Caucasus region, with little or no population separation [9], and a combination of SSRs and SNPs in Belgium identified two genetic pools, with one being overwhelmingly present [10]. Yet, the current study did not find that ISSR markers are especially useful in *Juniperus communis* var. *communis*, so an additional marker system (e.g., Sequence Characterized Amplified Region or Start Codon Targeted (SCoT) Polymorphisms) or identification of SNPs in nuclear and chloroplast regions is necessary to better assess the genetic diversity of this conifer.

The Mantel test was also used to assess the correlation between EO profiles and genetic data, accounting for geographic constraints (partial Mantel test). However, there was no geographic structuring of populations (on either chemophenetic or molecular data), nor was there concordance between molecular and chemophenetic markers. This is most likely due to different evolutionary constraints: ISSR markers are presumably selection-neutral, while essential oil profiles are not. Previous investigations of this taxon in the northwestern Balkans regarding the variability of leaf essential oil [4] have shown that environmental influences are not large, though they cannot be entirely excluded. For example, as shown in populations from Croatia, geographically distinct populations that grow on different parent rock and soil do not show significant differences in leaf essential oil composition. Additionally, in the same study, the authors showed that the leaf EO composition does not change substantially between years, though it does show some variability. Other authors have shown differences in grazing preference among chemotypes of common juniper leaf EOs [17]. Additional environmental interactions could, in fact, alter, in the long run, the average EO profile in a population through natural selection. ISSRs, presumably selection-neutral, suggest that there is constant gene flow between Balkan populations. This is not unexpected, since common juniper is a wind-pollinated, bird-dispersed species. Additional molecular markers or newer sequencing techniques (e.g., NGS) are necessary to identify single-nucleotide polymorphisms (SNPs) that are under similar constraints as terpenes. This would provide a more detailed insight into the genetic structuring of these populations and a better understanding of leaf EO as chemophenetic markers.

## 4. Materials and Methods

### 4.1. Plant Material

Branchlets of *J. c.* var. *communis* 10 cm long with two-year leaves were collected in late summer-early autumn from adult individuals from the central Balkans, at a height of about 75–100 cm. The branchlets were packed in PP zip bags, frozen in the field, and stored at −18 °C in the freezer until EO extraction. A total of 134 individuals from eight populations were collected. The plant material was identified by Nemanja Rajčević and Petar D. Marin. Digital vouchers were prepared for each individual, and herbarium voucher for each population was deposited in the Belgrade University Herbarium (BEOU), cf. Table 3.

### 4.2. Essential Oil Extraction

Leaves were separated from the branches and homogenized in a laboratory mill (Warring, Beverly, MA, USA) for 30 s before EO extraction. The homogenized material of each sample (ca. 3 g fresh weight) was subjected to 2 h simultaneous distillation and extraction in a Likens-Nickerson type apparatus using 5 mL of dichloromethane (CH_2_Cl_2_) [5]. The obtained extracts (0.5 mL) were stored in amber vials at 4 °C until further analysis.

### 4.3. Chemical Analysis

The EO composition was analysed using an Agilent 7890A apparatus with a 5975C mass-selective detector (Agilent, Santa Clara, CA, USA), flame ionization detector, and DB-5 MS fused-silica gel cap (30 m, 0.25 mm i.d., film thickness 0.25 mm). The oven temperature was programmed linearly from 60 °C to 240 °C at 3 °C/min, with the following parameters: injector temperature 220 °C; detector temperature 300 °C; transfer line temperature 240 °C; carrier gas, He (1.0 mL/min at 210 °C, with constant pressure); injection volume 1 μL; and split ratio, 20:1. Electronimpact mass spectra (EI-MS; 70 eV) were acquired over the *m*/*z* range 30–550. In all experiments, the relative amounts of volatile components were expressed as percentages of the total ion chromatogram peak area. Values under 0.05% were not considered during compound identification. Library search and mass spectral deconvolution and extraction were performed using the software NIST AMDIS version 2.64.113.71, with the linear retention index (RI) calibration data analysis parameters set to a “strong” level and a 10% penalty for compounds without RI. The search was performed against our in-house library, which contains 4972 spectra. The relative percentages of the identified compounds were calculated from their GC peak areas. The LRIs were experimentally determined using the standard method involving retention times (t_rs_) of *n*-alkanes, which were injected after the essential oil under the same chromatographic conditions. The linear RI was calculated for all compounds using the following formula: LRI = 100 × (t_rs_ − t_rn_)/(t_rn_ + 1 − t_rn_) + 100 × n [3].

### 4.4. DNA Extraction

DNA was extracted from silica-dried leaves (ca. 25 mg) following the protocol described previously [23]. The quality and concentration of each of the 73 DNA samples were assessed using NanoPhotometer N50 (IMPLEN, Munich, Germany). All isolates were amplified using five ISSR markers (Table 1). These markers were chosen for their informativeness in *Juniperus oxycedrus* L. [24].

### 4.5. PCR Amplification and Electrophoresis

PCR reactions were performed in a 12 μL reaction volume containing 6 μL of FastGene^®^ Optima HotStart ReadyMixFast (Nippon Genetics, Europe GmbH, Düsseldorf, Germany), 0.96 μL of each primer (0.8 μM) (Macrogen, Seoul, Republic of Korea), and 1 μL of *J. communis* DNA (100 ng final DNA concentration). The PCR amplifications were carried out on a thermal cycler (Eppendorf Mastercycler nexus GSX1, Thermo Fisher Scientific, Waltham, MA, USA) according to the following conditions: initial denaturation at 94 °C for 5 min, after which three cycles of 94 °C for 0.5 min, 1 min at melting temperature (Tm) (depending on the primer used, Table 1), and 72 °C for 2 min. In the subsequent 37 cycles, denaturation was 94 °C for 0.5 min, annealing was 1 min at annealing temperature (T_A_ = Tm − 5 °C) (depending on the primer used), followed by 2 min extension at 72 °C. In the end, a final extension was carried out at 72 °C for 10 min, after which the samples were kept at 4 °C until further investigation. The PCR products 3 μL of each sample were separated by electrophoresis on 3% (*w*/*v*) agarose gel (Cleaver Scientific Ltd., Rugby, UK) in 0.5× TBE buffer, stained with Midori Green Advanced DNA Stain (Nippon Genetics Europe GmbH, Düren, Germany). The length of PCR products was assessed using a GeneRuler 1 kb ladder (Thermo Fisher Scientific, Waltham, MA, USA) and then visualized under a Vilber Lourmat ECX-F20.M transilluminator (Collégien, France). Obtained gels were analysed using GelAnalyzer 23.1.1 (available at www.gelanalyzer.com) by Istvan Lazar Jr., PhD, and Istvan Lazar Sr., PhD, CSc.

### 4.6. Environmental Data

Bioclimatic data for the 30-year temperature and precipitation of all studied localities were obtained from the WorldClim 2.0 set of global climate layers, with a precision of approx. 1 km resolution [25]. Data was extracted using QGIS software 3.6 (QGIS Development Team, 2015).

### 4.7. Statistical Analysis

For phytochemical data, means, standard deviations, and distributions were checked before univariate (Mixture analysis) and multivariate analyses. Raw data of 18 compounds with relative abundance above 0.5% that had normal distribution and were not correlated were used in multivariate statistical analyses (Principal Components Analysis—PCA, Discriminant Analysis—DA, Multivariate ANOVA—MANOVA). Simple linear correlation (Pearson’s correlation) and multivariate correlation (Mantel and partial Mantel tests) were used to analyse correlations among individual components, groups of compounds, chemotypes, and bioclimatic data. All statistical analyses were performed using PAST 5.02 [26].

For molecular data, the amplified fragments were scored as 1 (present) or 0 (absent). Only strong, unambiguous bands were used for scoring. The efficiency of each marker in producing polymorphic DNA bands was expressed as the total number of bands, the number of polymorphic bands, the percentage of polymorphic bands, and the polymorphism information content (PIC) [27]. AMOVA (Analysis of Molecular Variance) and the population structure, based on the squared Euclidean distance matrix using Fst-analogue independent of ploidy level and the breeding system, was calculated using GenoDive v.3.0 [28,29,30]. Bayesian model-based estimation of population structure was performed using Structure version 2.3.4 [31]. The analyses were performed under the admixture model, assuming independent allele frequencies, with a burn-in period of 10,000, followed by 5000 Markov Chain Monte Carlo steps. The most likely value of K was determined using the method of Evanno et al. [31]. Principal coordinate analysis (PCoA) was performed to illustrate overall similarity among individuals using PastPAST 5.02 [26], based on Jaccard’s distance between all pairs of ISSR phenotypes. The Mantel test was used to assess the correlation between genetic (Nei’s genetic distances among populations) and geographic distances.

## 5. Conclusions

Chemophenetic and molecular markers indicate population differentiation and high genetic diversity within the studied populations. While essential oils highlight the geographic origin of the populations, ISSR markers suggested three putative genetic pools and the continuous exchange of genetic material between central Balkan populations. These results show that the central Balkan populations are a rich source of sabinene, an important monoterpene used in flavourings, perfume additives, and advanced biofuels, that is commercially available only from plant material. The present results call for additional molecular analysis to help identify the genotypes of sabinene-producing individuals and establish parameters for the geographic identification of the obtained plant material.

## Figures and Tables

**Table 1:** Chemical composition of leaf essential oil obtained from eight populations of *Juniperus communis* L. var. *communis* from the Balkans.

			Serbia			N. Macedonia		Greece		
#	Locality	LRI ^a^	Deliblato ^b^ (n = 29)	Mt. Kopaonik (n = 21)	Mt. Suva Planina (n = 24)	Mavrovo (n = 15)	Kozjak (n = 3)	Mt. Olympus (n = 20)	Vitos (n = 14)	Mt. Pindus (n = 8)
1	(2*Z*)-Hexenal	838	0.4 ± 0.2	0.3 ± 0.2	tr	tr	-	0.2 ± 0.1	tr	0.2 ± 0.1
2	(2*E*)-Hexenal	869	1.3 ± 0.6	0.8 ± 0.5	0.3 ± 0.2	tr	0.2 ± 0.2	tr	tr	tr
3	*α*-Thujene	924	1.9 ± 1	2.5 ± 0.9	1.3 ± 1	4.3 ± 1.9	0.6 ± 0.5	1.4 ± 1.1	1.8 ± 1.1	2.2 ± 0.4
4	*α*-Pinene	933	27.6 ± 15.1	17.3 ± 7.9	33.5 ± 18.0	22.8 ± 11.8	12.6 ± 7.5	21.1 ± 11.6	27.1 ± 13.8	17.9 ± 3.9
5	Camphene	946	0.2 ± 0.1	0.2 ± 0.3	0.2 ± 0.1	0.2 ± 0.1	tr	tr	0.2 ± 0.1	0.2 ± 0
6	Verbenene	961	tr	tr	0.2 ± 0.2	0.1 ± 0.2	-	tr	-	-
7	Sabinene	973	24.6 ± 12.8	35.9 ± 12.5	19.3 ± 15.8	18.8 ± 9.8	12.5 ± 6.5	15.5 ± 11.6	18.8 ± 10.1	27.1 ± 5.4
8	*β*-Pinene	976	1.4 ± 0.3	1.2 ± 0.3	1.7 ± 0.3	1.3 ± 0.5	1.3 ± 0.7	1.3 ± 0.4	1.5 ± 0.6	1.8 ± 0.4
9	Myrcene	988	3.8 ± 0.8	4 ± 0.9	2.9 ± 0.9	1.6 ± 0.7	2.1 ± 1.1	2.5 ± 0.8	2.9 ± 0.9	3.7 ± 0.7
10	*δ*2-Carene	1001	tr	tr	0.2 ± 0.2	tr	-	tr	tr	0.2 ± 0.1
11	*α*-Phelandrene	1004	0.4 ± 0.3	0.5 ± 0.5	0.5 ± 0.4	tr	-	0.4 ± 0.4	0.3 ± 0.3	0.6 ± 0.4
12	*δ*3-Carene	1010	0.3 ± 0.6	tr	0.7 ± 1.2	0.6 ± 1.7	1 ± 1.6	0.4 ± 0.9	0.2 ± 0.1	tr
13	*α*-Terpinene	1014	0.7 ± 0.3	0.8 ± 0.3	0.4 ± 0.3	0.8 ± 0.4	0.3 ± 0.1	0.7 ± 0.4	0.8 ± 0.5	0.8 ± 0.2
14	*p*-Cymene	1021	0.4 ± 0.2	0.3 ± 0.2	0.2 ± 0.2	1.6 ± 0.6	0.3 ± 0	1.2 ± 0.5	1 ± 0.7	1.3 ± 0.5
15	Limonene	1026	4.0 ± 2.0	6.2 ± 9.2	4.9 ± 4.1	4.3 ± 3.7	3.1 ± 1.4	7.3 ± 4.6	4.2 ± 1.4	6.1 ± 2.2
16	(*E*)-*β*-Ocimene	1044	0.2 ± 0.2	tr	tr	-	-	0.1 ± 0.2	tr	tr
17	*γ*-Terpinene	1055	1.4 ± 0.7	1.7 ± 0.7	0.8 ± 0.7	1.4 ± 0.6	0.9 ± 0.3	1.2 ± 0.7	1.5 ± 1	1.4 ± 0.3
18	*cis*-Sabinene hydrate	1064	0.3 ± 0.1	0.4 ± 0.2	0.2 ± 0.2	0.7 ± 0.3	0.6 ± 0.1	0.8 ± 0.6	0.5 ± 0.3	0.5 ± 0.1
19	Terpinolene	1086	1.5 ± 0.6	1.9 ± 0.5	1.2 ± 0.6	0.6 ± 0.3	1.3 ± 0.2	0.9 ± 0.4	1.1 ± 0.5	1.1 ± 0.3
20	*trans*-Sabinene hydrate	1097	0.3 ± 0.1	0.4 ± 0.2	0.2 ± 0.2	0.9 ± 0.5	0.6 ± 0.3	0.7 ± 0.3	0.5 ± 0.3	0.6 ± 0.2
21	Linalool	1102	-	-	0.1 ± 0.2	0.1 ± 0.2	0.2 ± 0.3	-	0.1 ± 0.3	-
22	2-Methyl butyl isovalerate	1102	-	-	-	-	0.2 ± 0.1	-	0.2 ± 0.1	0.2 ± 0.1
23	*cis*-Thujone	1108	-	-	-	-	-	0.2 ± 0.1	-	-
24	*trans*-Thujone	1115	-	tr	-	tr	tr	0.2 ± 0.1	0.2 ± 0.1	0.2 ± 0.1
25	*cis-p*-Menth-2-en-1-ol	1119	0.2 ± 0.1	0.2 ± 0.1	tr	0.3 ± 0.1	0.2 ± 0	0.3 ± 0.2	0.3 ± 0.2	0.3 ± 0.1
26	*α*-Campholenal	1126	-	-	-	0.3 ± 0.3	tr	0.2 ± 0.3	0.2 ± 0.2	tr
27	*trans*-Pinocarveol	1134	-	-	-	0.9 ± 0.9	-	0.5 ± 0.3	0.3 ± 0.3	-
28	*trans-p*-Menth-2-en-1-ol	1136	tr	tr	tr	-	tr	-	tr	0.3 ± 0.3
29	*trans*-Sabinol	1140	-	0.1 ± 0.2	-	-	-	0.1 ± 0.3	-	-
30	*trans*-Verbenol	1142	-	-	0.1 ± 0.3	1.3 ± 1.6	0.3 ± 0.4	0.9 ± 1.1	0.6 ± 0.9	0.2 ± 0.3
31	Sabina ketone	1153	-	-	-	0.3 ± 0.2	-	0.1 ± 0.2	tr	tr
32	Pinocarvone	1158	-	-	-	tr	-	0.3 ± 0.2	0.3 ± 0.2	0.1 ± 0.2
33	Borneol	1163	-	-	-	tr	-	0.3 ± 0.2	0.2 ± 0.2	0.1 ± 0.2
34	*p*-Mentha-1,5-dien-8-ol	1165	-	-	-	0.5 ± 0.8	0.1 ± 0.2	0.2 ± 0.4	0.1 ± 0.2	tr
35	3-Thujanol	1167	-	-	-	0.2 ± 0.1	-	tr	-	-
36	Terpinen-4-ol	1176	2.5 ± 1.3	3.1 ± 1.3	1.5 ± 1.2	3.2 ± 1.5	3 ± 0.2	4.6 ± 1.9	4.2 ± 2.6	3.7 ± 1.1
37	*p*-Cymen-8-ol	1183	tr	tr	-	0.6 ± 0.3	0.2 ± 0.1	0.4 ± 0.3	0.3 ± 0.2	0.3 ± 0.2
38	Cryptone	1185	-	-	-	-	-	0.1 ± 0.2	-	-
39	*α*-Terpineol	1188	0.2 ± 0.1	0.3 ± 0.2	0.2 ± 0.2	0.4 ± 0.3	0.5 ± 0.3	0.9 ± 0.4	0.6 ± 0.2	0.7 ± 0.5
40	Myrtenol	1195	-	-	tr	tr	-	0.5 ± 0.4	tr	-
41	Myrtenal	1196	-	-	-	0.4 ± 0.5	-	-	0.3 ± 0.2	0.1 ± 0.2
42	Verbenone	1207	-	-	-	0.5 ± 0.8	0.1 ± 0.2	0.5 ± 0.7	0.3 ± 0.5	0.2 ± 0.2
43	*trans*-Carveol	1216	-	0.1 ± 0.2	-	0.2 ± 0.2	-	0.2 ± 0.4	tr	-
44	Citronellol	1226	-	-	-	-	-	0.3 ± 0.6	tr	-
45	*cis*-*p*-Mentha-1(7),8-dien-2-ol	1128	-	-	-	-	-	0.1 ± 0.2	-	-
46	(*E*)-Cinnamaldehyde	1270	-	-	-	-	-	1.1 ± 0.9	0.9 ± 0.5	1.2 ± 1.6
47	*α*-Terpinen-7-al	1283	-	-	-	-	-	tr	-	-
48	Bornyl acetate	1284	0.3 ± 0.1	0.2 ± 0.1	0.3 ± 0.2	0.2 ± 0.2	0.7 ± 0.4	0.3 ± 0.2	0.3 ± 0.2	0.2 ± 0.1
49	Myrtenyl acetate	1322	-	tr	0.2 ± 0.2	-	0.2 ± 0.1	-	-	-
50	*α*-Terpinyl acetate	1347	0.2 ± 0.4	-	0.4 ± 0.6	-	-	-	-	-
51	*α*-Cubebene	1349	-	0.4 ± 0.6	-	0.4 ± 0.6	tr	0.2 ± 0.5	0.1 ± 0.2	0.1 ± 0.3
52	*α*-Copaene	1374	0.2 ± 0.3	0.2 ± 0.2	0.6 ± 0.7	0.6 ± 0.6	0.5 ± 0.7	0.5 ± 0.4	1.1 ± 0.4	1.1 ± 0.6
53	*β*-Bourbonene	1383	tr	tr	0.2 ± 0.1	tr	-	-	0.2 ± 0.1	-
54	*β*-Elemene	1391	0.4 ± 0.2	0.3 ± 0.2	0.6 ± 0.6	1.1 ± 0.6	1.8 ± 1.5	1.3 ± 1	1.6 ± 0.9	0.7 ± 0.6
55	Sibirene	1401	-	-	0.5 ± 0.9	0.4 ± 0.7	-	0.3 ± 0.4	0.4 ± 0.4	0.4 ± 0.3
56	(*E*)-Caryophyllene	1418	1.1 ± 1.2	1 ± 1.2	1.3 ± 1.1	1.3 ± 1.6	2.8 ± 2.8	2.7 ± 2.6	1.9 ± 1.1	2.5 ± 1.8
57	*β*-Copaene	1428	-	-	-	tr	-	tr	0.2 ± 0.1	-
58	*cis*-Thujopsene	1430	0.7 ± 2	-	-	0.4 ± 1.4	tr	-	-	-
59	*γ*-Elemene	1433	0.3 ± 1.5	-	0.1 ± 0.2	-	2.8 ± 2.3	0.8 ± 0.8	0.7 ± 1.1	0.2 ± 0.2
60	*α*-Humulene	1452	0.8 ± 0.8	0.8 ± 0.7	1 ± 0.7	1.1 ± 1.1	1.9 ± 1.8	1.9 ± 1.8	1.5 ± 0.8	1.6 ± 1.4
61	(*E*)-*β*-Farnesene	1456	-	-	-	-	-	-	-	0.3 ± 0.7
62	*trans*-Cadina-1(6),4-diene	1473	tr	tr	tr	-	tr	-	tr	tr
63	*γ*-Muurolene	1476	0.3 ± 0.3	0.7 ± 1.7	0.2 ± 0.1	0.6 ± 0.2	0.3 ± 0.2	1.1 ± 1.3	0.2 ± 0.1	tr
64	Germacrene D	1481	3.5 ± 2.3	2.8 ± 1.9	5.6 ± 3.2	1.8 ± 1.5	2.7 ± 1.1	2 ± 2.2	3.4 ± 1.4	2 ± 2.2
65	ar-Curcumene	1483	-	-	-	0.2 ± 0.6	0.9 ± 1.6	0.2 ± 0.9	-	2.1 ± 2.9
66	*β*-Selinene	1486	0.3 ± 0.2	0.2 ± 0.1	0.2 ± 0.2	1 ± 0.5	0.4 ± 0.3	0.2 ± 0.1	0.2 ± 0.1	tr
67	*trans*-Muurola-4(14),5-diene	1492	tr	tr	tr	tr	tr	-	0.3 ± 0.2	tr
68	*epi*-Cubebol	1493	-	-	0.1 ± 0.5	0.6 ± 0.6	-	0.2 ± 0.2	-	-
69	*α*-Selinene	1494	-	-	-	0.1 ± 0.4	-	-	-	-
70	*β*-Alaskene	1497	1.0 ± 0.6	0.6 ± 0.5	-	-	-	-	-	-
71	Bicyclogermacrene	1498	-	0.2 ± 0.4	0.9 ± 0.4	0.1 ± 0.4	0.8 ± 0.4	0.3 ± 0.3	0.3 ± 0.3	0.6 ± 0.3
72	*α*-Muurolene	1501	0.5 ± 0.3	0.3 ± 0.2	0.5 ± 0.7	0.3 ± 0.1	0.6 ± 0.4	0.2 ± 0.1	0.2 ± 0.1	0.3 ± 0.2
73	Cuparene	1504	-	-	-	0.1 ± 0.2	-	-	-	-
74	Germacrene A	1506	tr	0.9 ± 0.5	-	-	-	-	-	-
75	Premnaspirodiene	1506	1.2 ± 0.7	-	1.1 ± 0.7	-	0.8 ± 0.6	-	-	0.2 ± 0.2
76	*β*-Bisabolene	1510	-	-	-	-	tr	-	-	-
77	*γ*-Cadinene	1514	0.8 ± 0.7	0.6 ± 0.9	0.7 ± 0.7	0.9 ± 0.4	-	1.2 ± 0.9	0.5 ± 0.5	-
78	Cubebol	1515	-	-	-	tr	-	-	-	0.6 ± 0.3
79	*δ*-Cadinene	1524	-	-	-	1.3 ± 1.3	2.7 ± 3.1	-	2.2 ± 0.6	2.2 ± 1.3
80	*γ*-Cuprenene	1533	0.2 ± 0.3	-	-	tr	-	-	-	-
81	*trans*-Cadina-1,4-diene	1534	-	-	-	-	0.2 ± 0.2	-	-	tr
82	*α*-Cadinene	1537	tr	tr	tr	tr	0.2 ± 0.1	tr	-	-
83	Elemol	1552	tr	-	tr	tr	0.3 ± 0.3	tr	tr	tr
84	Selina-3,7(11)-diene	1559	-	-	2.6 ± 2.7	-	-	-	-	-
85	Germacrene B	1560	2.9 ± 2.6	1.9 ± 1.5	0.1 ± 0.3	0.6 ± 0.8	7.1 ± 9.2	0.3 ± 0.3	0.3 ± 0.4	0.6 ± 0.5
86	(*E*)-Nerolidol	1565	tr	-	0.5 ± 1.5	0.1 ± 0.2	0.6 ± 0.9	0.4 ± 0.5	0.3 ± 0.2	0.4 ± 0.5
87	Germacrene D-4-ol	1578	1.4 ± 1.1	0.2 ± 0.5	0.5 ± 2.1	0.7 ± 1.0	0.3 ± 0.6	1.0 ± 1.1	1.3 ± 0.9	0.5 ± 0.5
88	Spathulenol	1579	0.2 ± 0.5	1.0 ± 0.9	1.6 ± 1.4	1.5 ± 1.1	3.2 ± 3.4	0.5 ± 0.6	0.3 ± 0.3	0.6 ± 1
89	Caryophyllene oxide	1582	-	-	-	1.9 ± 3.3	0.8 ± 1	-	0.6 ± 0.4	0.7 ± 0.6
90	*α*-Eudesmol	1652	0.2 ± 0.3	0.2 ± 0.4	tr	-	-	1 ± 0.9	-	-
91	*β*-Copaen-4-*α*-ol	1588	-	-	-	tr	-	-	tr	0.4 ± 0.9
92	Cedrol	1601	0.2 ± 0.4	-	-	0.9 ± 0.5	0.3 ± 0.4	0.2 ± 0.3	-	tr
93	Humulene epoxide II	1607	-	tr	-	1.4 ± 1.8	0.2 ± 0	0.6 ± 0.5	0.5 ± 0.3	0.5 ± 0.5
94	*β*-Oplopenone	1610	tr	-	tr	0.3 ± 0.2	-	0.2 ± 0.4	-	-
95	1-*epi*-Cubenol	1631	tr	tr	0.4 ± 0.6	0.5 ± 0.5	0.3 ± 0.2	0.3 ± 0.2	0.4 ± 0.3	0.4 ± 0.2
96	*epi*-*α*-Murrolol (=tau-muurolol)	1646	1.1 ± 0.6	0.8 ± 0.6	0.9 ± 0.5	0.5 ± 0.4	1.8 ± 1.7	0.9 ± 0.6	0.9 ± 0.3	0.7 ± 0.3
97	*α*-Muurolol (=torreyol)	1649	0.2 ± 0.1	tr	0.2 ± 0.1	tr	0.3 ± 0.4	0.2 ± 0.1	0.2 ± 0.1	0.5 ± 0.4
98	3-Thujopsanone	1650	-	-	-	0.1 ± 0.2	-	-	-	-
99	*α*-Cadinol	1660	1.8 ± 1.1	1.3 ± 1.2	1.3 ± 0.9	0.9 ± 0.8	2.7 ± 3	1.3 ± 1	1.3 ± 0.7	0.3 ± 0.7
100	14-hydroxy-9-*epi*-(*E*)-Caryophyllene	1674	-	-	-	0.3 ± 0.7	tr	0.2 ± 0.1	0.2 ± 0.1	-
101	Germacra-4(15),5,10 (14)-trien-1-*α*-ol	1685	-	tr	-	0.2 ± 0.1	0.1 ± 0.2	0.3 ± 0.1	0.2 ± 0.1	-
102	Shyobunol	1695	0.2 ± 0.2	-	0.2 ± 0.2	0.3 ± 0.3	-	tr	-	-
103	Eudesm-7(11)-en-4-ol	1700	-	0.2 ± 0.2	-	-	0.2 ± 0.2	-	0.2 ± 0.1	tr
104	Amorpha-4,9-dien-2-ol	1705	-	-	-	0.1 ± 0.2	-	0.2 ± 0.1	-	-
105	(2*Z*,6*Z*)-Farnesal	1718	-	0.1 ± 0.2	0.1 ± 0.3	0.2 ± 0.2	-	0.6 ± 0.7	0.3 ± 0.1	-
106	(2*Z*, 6*E*)-Farnesol	1725	-	-	-	-	1.4 ± 2.4	-	-	-
107	*iso*-Longifolol	1729	-	-	-	-	0.3 ± 0.6	-	-	-
108	Oplopanone	1740	-	-	-	0.2 ± 0.1	0.1 ± 0.2	tr	-	-
109	Hexadecanoic acid	1960	-	-	-	-	tr	0.4 ± 0.3	tr	-
110	Manoyl oxide	1995	-	0.2 ± 0.5	-	0.5 ± 0.4	2.5 ± 4.1	1 ± 2.5	0.2 ± 0.2	tr
111	Phyllocladene	2019	tr	tr	-	-	0.2 ± 0.1	tr	-	-
112	Abietatriene	2059	0.2 ± 0.2	0.2 ± 0.3	tr	0.3 ± 0.2	-	0.2 ± 0.4	-	-
113	Abietadiene	2070	0.1 ± 0.2	0.5 ± 1.3	0.2 ± 0.3	tr	0.7 ± 0.6	0.6 ± 0.5	0.2 ± 0.1	0.4 ± 0.3
114	Sandaracopimarinal	2182	tr	tr	-	0.1 ± 0.3	0.2 ± 0.1	tr	tr	tr
115	*trans*-Totarol, methyl ether	2232	tr	-	0.1 ± 0.2	0.2 ± 0.3	0.6 ± 0.8	0.3 ± 0.3	0.2 ± 0.2	0.2 ± 0
116	dehydro-Abietal	2274	-	-	-	-	0.6 ± 0.8	0.2 ± 0.2	tr	0.2 ± 0.2
117	Abietal	2323	tr	0.2 ± 0.3	0.2 ± 0.6	0.1 ± 0.2	2.4 ± 3.8	1.3 ± 0.8	0.6 ± 0.4	0.9 ± 0.6
118	Abietol	2406	-	-	-	-	0.2 ± 0.3	0.2 ± 0.2	tr	-
**Total monoterpenes**			**73.2 ± 1.0**	**79.1 ± 1.0**	**72.2 ± 1.3**	**70.7 ± 1.0**	**43.8 ± 0.6**	**69.8 ± 1.1**	**73.2 ± 1.0**	**74.2 ± 0.5**
*Monoterpene hydrocarbons*			*67.1 ± 1.9*	*70.9 ± 1.9*	*66.9 ± 2.4*	*58.1 ± 1.8*	*35.0 ± 1.1*	*53.6 ± 1.9*	*60.7 ± 1.7*	*63.6 ± 0.8*
*Oxygenated monoterpenes*			*6.1 ± 0.1*	*8.2 ± 0.1*	*5.3 ± 0.1*	*12.6 ± 0.2*	*8.8 ± 0.1*	*16.2 ± 0.3*	*12.5 ± 0.2*	*10.6 ± 0.1*
**Total sesquiterpenes**			**21.1 ± 0.2**	**15.7 ± 0.2**	**23.1 ± 0.3**	**24.9 ± 0.3**	**45.0 ± 0.6**	**22.7 ± 0.3**	**23.3 ± 0.2**	**22.1 ± 0.2**
*Sesquiterpene hydrocarbons*			*15.1 ± 0.4*	*11.3 ± 0.3*	*17.0 ± 0.3*	*12.5 ± 0.3*	*30.2 ± 0.7*	*13.5 ± 0.3*	*15.8 ± 0.2*	*16.6 ± 0.3*
*Oxygenated sesquiterpenes*			*6.0 ± 0.1*	*4.5 ± 0.1*	*6.1 ± 0.2*	*12.4 ± 0.3*	*14.8 ± 0.4*	*9.2 ± 0.2*	*7.5 ± 0.1*	*5.4 ± 0.1*
**Total diterpenes**			**0.9 ± 0.1**	**1.5 ± 0.2**	**1.0 ± 0.1**	**1.5 ± 0.1**	**8.8 ± 0.7**	**4.1 ± 0.3**	**1.5 ± 0.1**	**2.2 ± 0.1**
*Diterpene hydrocarbons*			*0.6 ± 0.1*	*1.0 ± 0.2*	*0.6 ± 0.1*	*0.6 ± 0.1*	*2.3 ± 0.2*	*1.0 ± 0.1*	*0.4 ± 0.0*	*0.7 ± 0.1*
*Oxygenated diterpenes*			*0.3 ± 0.0*	*0.5 ± 0.1*	*0.4 ± 0.1*	*0.9 ± 0.1*	*6.5 ± 1.3*	*3.1 ± 0.5*	*1.2 ± 0.1*	*1.4 ± 0.1*
Other ^c^			1.8 ± 0.2	1.0 ± 0.2	0.4 ± 0.1	0.2 ± 0.0	0.3 ± 0.1	0.3 ± 0.1	0.2 ± 0.0	0.3 ± 0.0
**TOTAL**			**97.0 ± 1.2**	**97.3 ± 1.4**	**96.6 ± 1.5**	**97.3 ± 1.8**	**98.0 ± 1.2**	**97.1 ± 1.7**	**98.2 ± 0.9**	**98.9 ± 0.3**
**Number of compounds**			**74–91**	**65–94**	**53–82**	**71–92**	**80–85**	**75–95**	**81–90**	**77–85**

^a^ LRI—experimental linear retention indices obtained by injecting a series of *n*-alkanes under the same chromatographic conditions using DB-5 MS fused-silica gel cap column; ^b^ mean values *±* standard deviation, tr—trace (0.05 < x < 0.1), n—number of individual samples; ^c^ non-terpenic volatiles, e.g., aliphatic and aromatic hydrocarbon.

**Table 2:** Marker parameters for each ISSR primer used with *Juniperus communis* L. var. *communis*.

ISSR	Primer Sequence	T_A_^1^	TB ^2^	PB ^3^	%PB ^4^	PIC ^5^
15	5′-ACA CAC ACA CAC ACA CG-3′	49.8	11	11	100%	0.334
12	5′-GAG AGA GAG AGA GAG AYT-3′	44.3	14	14	100%	0.275
14	5′-CTC TCT CTC TCT CTC TRA-3′	42.7	12	12	100%	0.249
13	5′-GAG AGA GAG AGA GAG AYC-3′	47.2	17	17	100%	0.308
10	5′-AGA GAG AGA GAG AGA CYT-3′	45.6	24	24	100%	0.318
**Total**			**78**	**78**	**100%**	**0.297**

^1^ T_A_—Annealing temperature (°C); ^2^ TB—Total bands; ^3^ PB—Polymorphic bands (single occurrence bands were not calculated); ^4^ %PB—Percentage of polymorphic bands; ^5^ PIC—values calculated according to [14].

**Table 3:** Geographic characteristics of the analysed populations of *Juniperus communis* var. *communis* (Cupressaceae) from the central Balkans and the number of samples per analysis.

Locality	Country	Latitude [N°]	Longitude [E°]	Altitude [m]	EO ^1^	ISSR ^2^	BEOU ^3^
Deliblato sands	Serbia	44.920	21.149	117	29	10	17,204
Mt. Kopaonik		43.312	20.914	1052	21	9	17,408
Mt. Suva planina		43.197	22.144	815	24	10	17,396
Mavrovo (Mt. Bistra)	North Macedonia	41.625	20.682	1849	15	10	17,216
Mt. Kozjak		41.888	21.229	718	3	10	18,056
Mt. Olympus	Greece	40.006	22.401	858	20	10	17,183
Vitos		40.206	21.128	867	14	9	18,050
Mt. Pindus		39.907	20.782	842	8	5	17,174

^1^ EO—Number of samples for essential oil analysis; ^2^ ISSR—number of samples for ISSR analysis; ^3^ University of Belgrade Faculty of Biology Herbarium (BEOU) voucher number.

## Data Availability

All data are available upon request to the authors.

## Supplementary Materials

The following supporting information can be downloaded at: https://www.mdpi.com/article/10.3390/plants15081266/s1, Figure S1: Principal Component Analysis (PCA) scatter plot of eighteen components of *Juniperus communis* L. var. *communis* leaf essential oil., Figures S2–S4: Gas chromatogram of identified chemotypes of *Juniperus communis* L. var. *communis* from central Balkans; Figure S5. Pearson’s correlation of 18 leaf essential oil components from *Juniperus communis* L. var. *communis* with longitude, latitude, altitude and bioclimatic parameters. Only values with *p* < 0.05 are presented. Size of the dot corresponds to the Pearson’s coefficient R.
