# Supplementary material for: Molecular and Phytochemical Variability of Common Juniper (Juniperus communis L.) in the Central Balkans Reveals Differentiation of Populations
Source: Plants (Basel). 2026 Apr 20;15(8):1266. doi: 10.3390/plants15081266 (PMC13120482; doi:10.3390/plants15081266)
Supplement: Supplementary file 1 [file plants-15-01266-s001.zip › plants-4247157-supplementary.pdf]

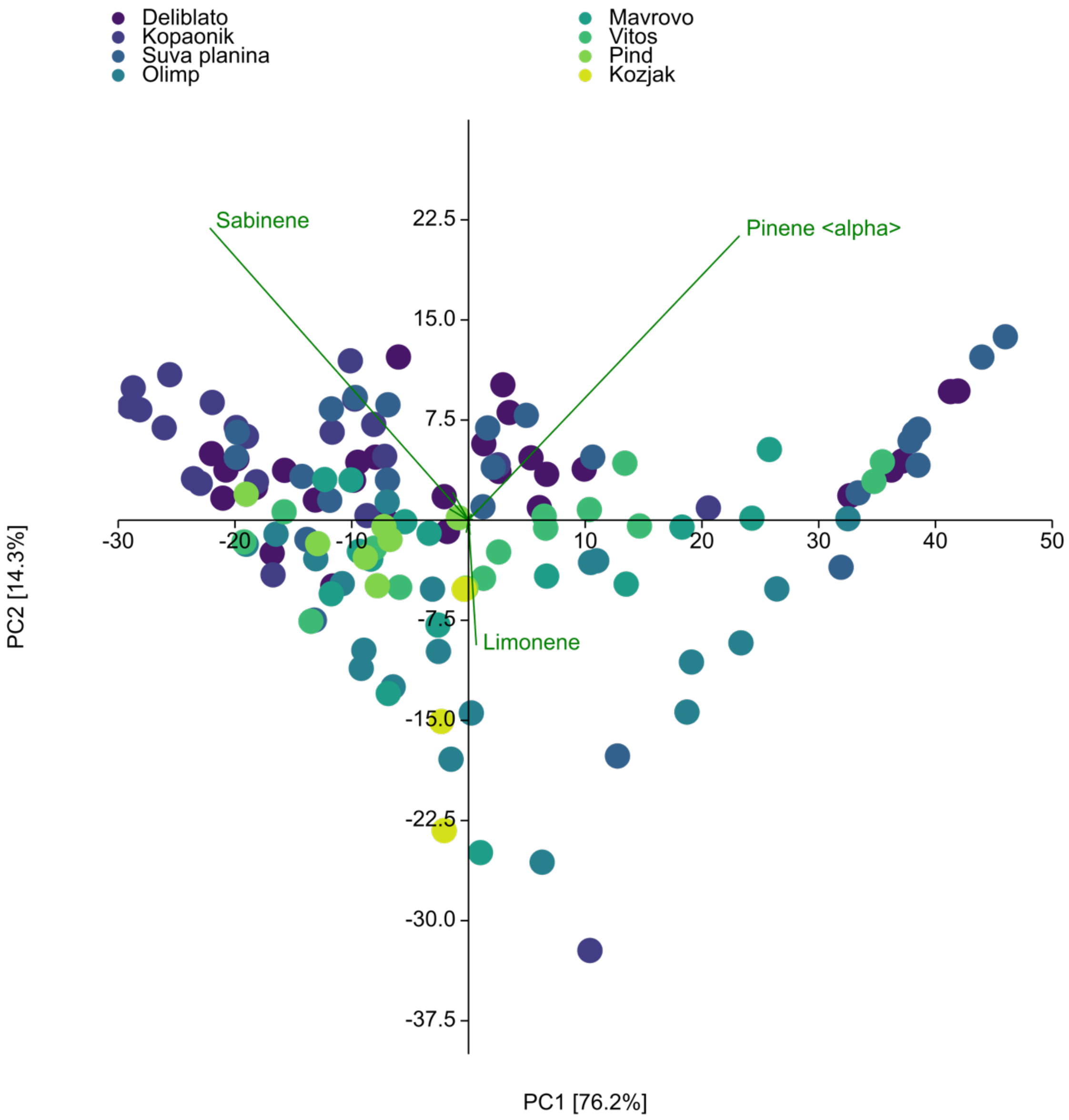

Supplementary Figure S1. Principal Component Analysis (PCA) scatter plot of eighteen components of *Juniperus communis* L. var. *communis* leaf essential oil.

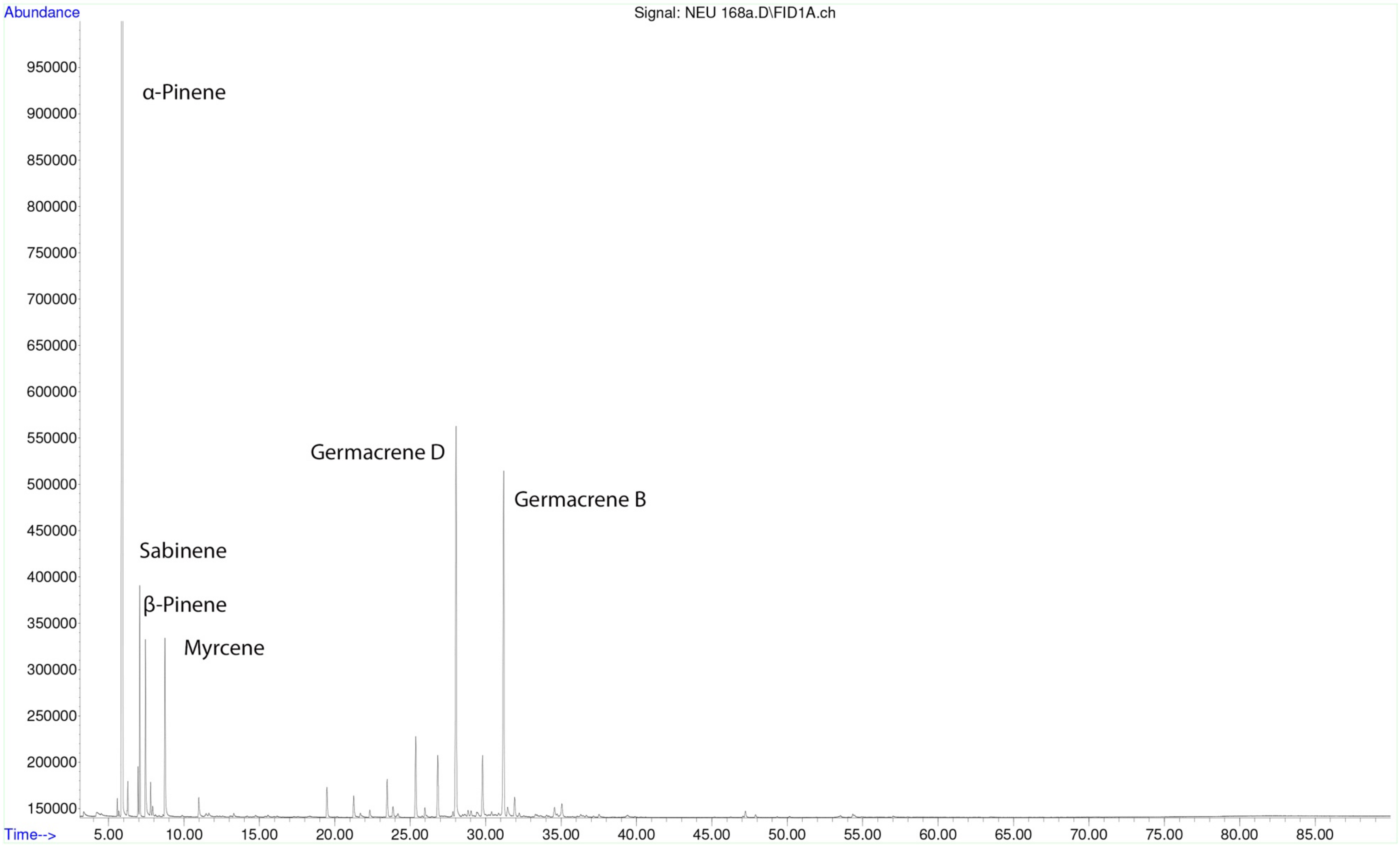

Supplementary Figure S2. Gas chromatogram of α-pinene chemotype of *Juniperus communis* L. var. *communis* from central Balkans.

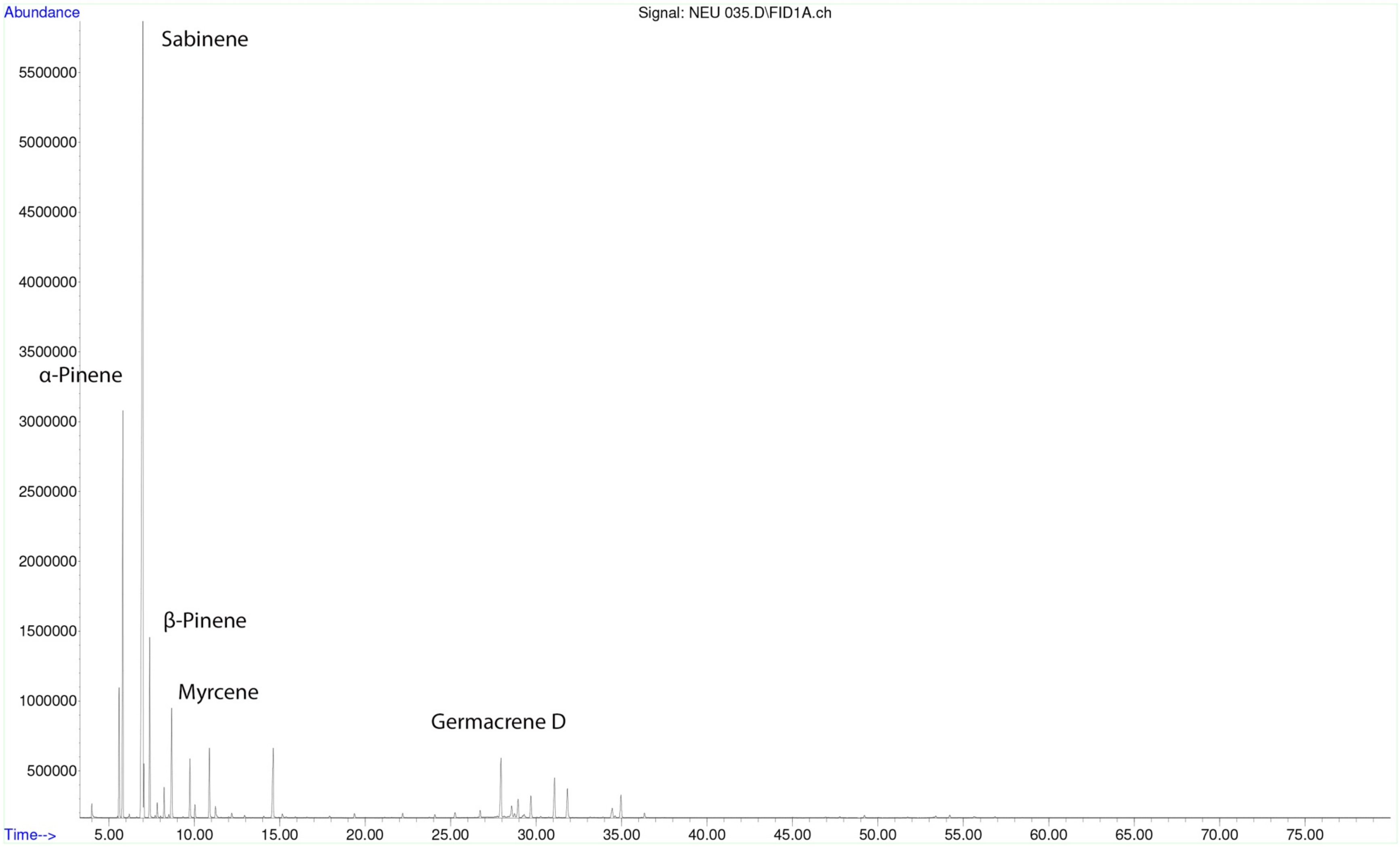

Supplementary Figure S3. Gas chromatogram of sabinene chemotype of *Juniperus communis* L. var. *communis* from central Balkans.

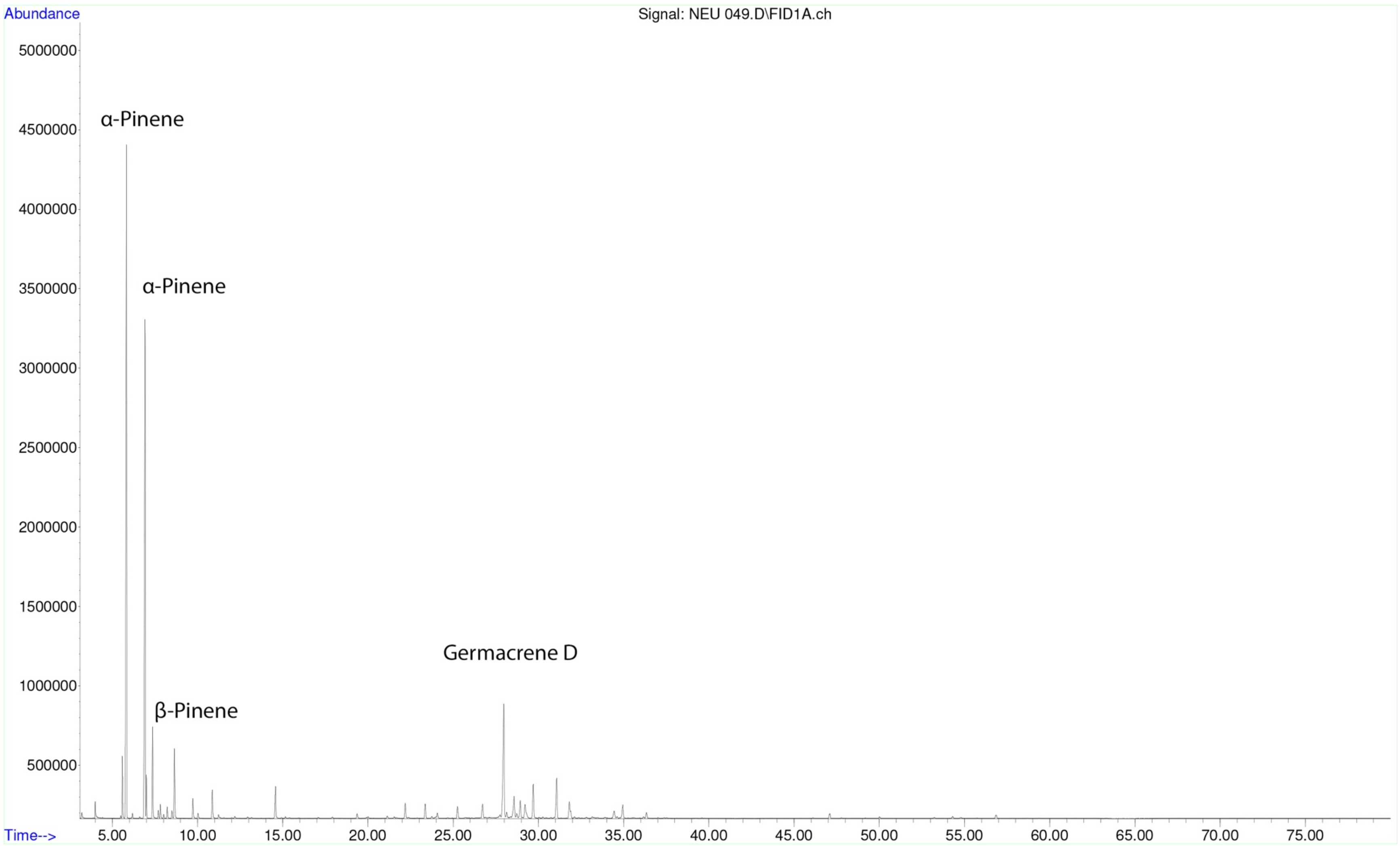

Supplementary Figure S4. Gas chromatogram of intermediate chemotype of *Juniperus communis* L. var. *communis* from central Balkans.

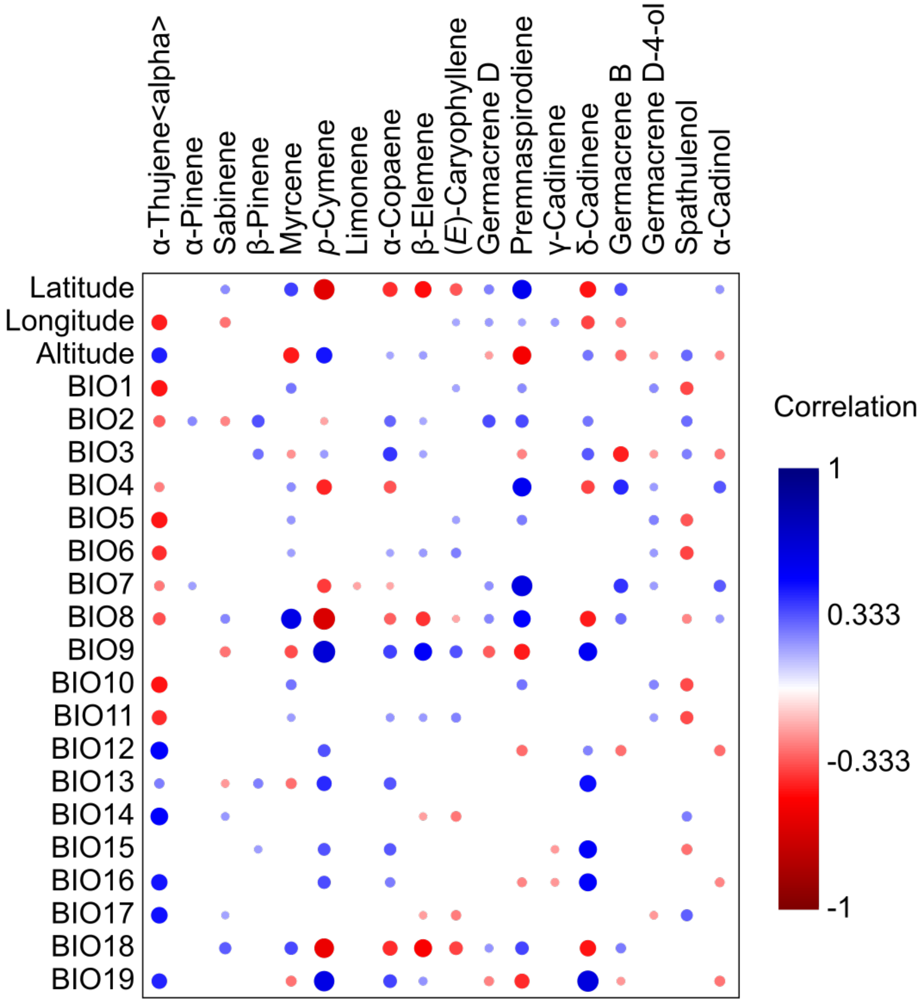

Supplementary Figure S5. Pearson's correlation of 18 leaf essential oil components from *Juniperus communis* L. var. *communis* with longitude, latitude, altitude and bioclimatic parameters. Only values with $p<0.05$ are presented. Size of the dot corresponds to the Pearson's coefficient R.
